# Physiological and psychological responses of young males during spring-time walks in urban parks

**DOI:** 10.1186/1880-6805-33-8

**Published:** 2014-05-01

**Authors:** Chorong Song, Harumi Ikei, Miho Igarashi, Masayuki Miwa, Michiko Takagaki, Yoshifumi Miyazaki

**Affiliations:** 1 Center for Environment, Health and Field Sciences, Chiba University, Chiba, Japan

**Keywords:** Urban green space, Walking, Spring, Physiological relaxation, Preventive medicine, Heart rate, Heart rate variability, Semantic differential method, Profile of Mood States, State-Trait Anxiety Inventory

## Abstract

**Background:**

It is widely believed that contact with the natural environment can improve physical and mental health. Urban green spaces may provide city residents with these benefits; however, there is a lack of empirical field research on the health benefits of urban parks.

**Methods:**

This field experiment was performed in May. Seventeen males aged 21.2 ± 1.7 years (mean ± standard deviation) were instructed to walk predetermined 15-minute courses in an urban park and a nearby city area (control). Heart rate and heart rate variability (HRV) were measured to assess physiological responses. The semantic differential (SD) method, Profile of Mood States (POMS), and State-Trait Anxiety Inventory (STAI) were used to measure psychological responses.

**Results:**

Heart rate was significantly lower while walking in the urban park than while walking in the city street. Furthermore, the urban park walk led to higher parasympathetic nervous activity and lower sympathetic nervous activity compared with the walk through the city street. Subjective evaluations were generally in accordance with physiological reactions, and significantly higher scores were observed for the ‘comfortable’, ‘natural’, and ‘relaxed’ parameters following the urban park walk. After the urban park walk, the score for the ‘vigor’ subscale of the POMS was significantly higher, whereas that for negative feelings such as ‘tension-anxiety’ and ‘fatigue’ was significantly lower. The score for the anxiety dimension of the STAI was also significantly lower after the urban park walk.

**Conclusions:**

Physiological and psychological results from this field experiment provide evidence for the physiological and psychological benefits of urban green spaces. A brief spring-time walk in an urban park shifted sympathetic/parasympathetic balance and improved mood state.

## Background

In recent years, the primary focus of healthcare has been shifting from the treatment of disease to health promotion, disease prevention, and improved quality of life. Natural environments such as urban green spaces may provide such benefits, promoting human health and well being. Indeed, many studies have demonstrated a significant positive relationship between exposure to natural environments and physical and mental health. Several questionnaire-based studies reported restorative effects against psychological stressors or mental fatigue [1-4] and improved mood and cognitive function [5-8]. Improved physiological measurement techniques have generated additional empirical evidence that time spent in a forest can decrease blood pressure [9-13] and pulse rate [9-15], suppress sympathetic nervous activity [9,11-15], increase parasympathetic nervous activity [9,11-15], decrease cortisol levels [10-16], and decrease cerebral blood flow in the prefrontal cortex [16]. These studies suggest that human beings are more relaxed in forested environments. Moreover, visiting a forested environment enhanced human natural killer cell activity and improved immune function [17], effects that lasted for approximately one month [18,19]. According to these previous studies, contact with nature brings about physiological and psychological relaxation effects and improves immune function, clearly demonstrating the preventive medical effects of nature [20].

In our modern urbanized society, however, opportunities for such interactions with nature are limited. Of late, the potential health benefits of urban green spaces have been studied. Urban green space can enhance the city environment by influencing temperature, wind, humidity, rainfall, soil erosion, flooding, air quality, scenic quality, and plant and animal diversity [21]. In addition, urban green space may provide important social and psychological benefits that enrich human life [22]. Recent demographic studies have found a positive association between exposure to urban green space and the perceived general health of residents [23,24]. Living in areas with walkable green spaces also increased the longevity of senior citizens, independent of age, sex, marital status, baseline functional status, and socioeconomic status [25].

Most individuals in industrialized countries live in urban areas and will continue to do so for the foreseeable future [26]; therefore, any beneficial effects of urban green space can improve general health and longevity. However, there is a lack of empirical research on the therapeutic benefits of urban green space [27]. Many investigators have emphasized the importance of field research [6,10] that can examine the effects of real environments.

We have conducted such a field experiment during winter [28], but there have been no such studies conducted during spring. Therefore, the aim of this study was to examine the physiological and psychological responses of young males during spring-time walks in urban parks.

## Methods

This field experiment was conducted in May 2013 in Kashiwanoha Park (hereafter referred to as the urban park) in Kashiwa City, Chiba Prefecture, Japan. A city area around the urban park (hereafter referred to as the city area) was selected as a control site.

Seventeen Japanese male university students aged 21.2 ± 1.7 years (mean ± standard deviation) participated in this experiment. Before the experiment, the subjects were fully informed about the aims and procedures involved. After receiving a description of the experiment, the subjects signed an agreement to participate. On the experimental day, alcohol and tobacco were prohibited. This study was performed according to the regulations of the Ethics Committee of the Center for Environment, Health and Field Sciences, Chiba University, Japan.

**Figure 1:**
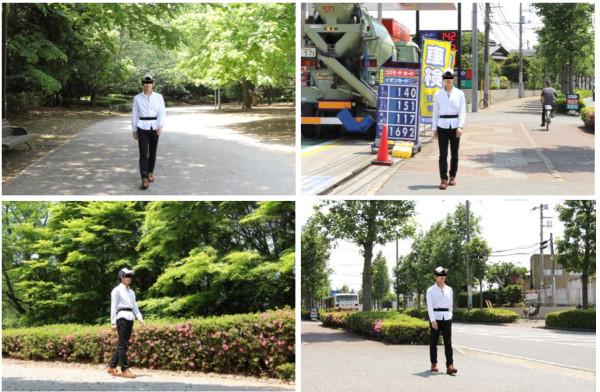
Experimental sites.

The subjects were instructed to walk predetermined courses through the urban park and the city area. Each course took about 15 minutes to complete. Subjects walked both courses on the same day (at 10 to 12 am and 1 to 3 pm), and the site order was counterbalanced across subjects. On the day of the experiment, the weather was sunny. A 15-minute walk was taken under direct sunlight in the city area at a temperature of 27.0 ± 1.7°C (mean ± standard deviation) and relative humidity of 37.3 ± 8.4% (mean ± standard deviation). In the urban park, the walk was taken under both direct sunlight and shade at a temperature of 24.7 ± 1.6°C and relative humidity of 39.2 ± 5.3%. The trees in the park had light green leaves, and the azaleas were in full bloom (Figure 1). There were no significant differences in baseline physiological indices or average walking speed between the two environments. After walking, the subjects returned to a waiting room and completed several questionnaires. They rested for approximately 20 minutes, after which they walked the other experimental course.

Heart rate and heart rate variability (HRV) were measured to assess cardiovascular and autonomic nervous system responses. The HRV was measured using a portable electrocardiograph (Activtracer AC-301A, GMS, Tokyo, Japan), and frequency spectra were generated using an HRV software tool (MemCalc/Win, GMS, Tokyo, Japan). For real-time HRV analysis using the maximum entropy method, interbeat (R-R) intervals were obtained continuously. In this study, two broad HRV spectral components were calculated: low frequency (LF; 0.04 to 0.15 Hz) and high frequency (HF; 0.15 to 0.40 Hz). The HF component is an estimate of parasympathetic nervous activity, while the LF/HF ratio is an estimate of sympathetic nervous activity [29,30]. To normalize HRV parameters across subjects, we used natural logarithmic transformed values for the analysis [31]. The heart rate and HRV data were collected at 1-minute intervals at each experimental location, and the 15-minute average was compared between sites.

Three different questionnaires were used to investigate psychological responses after walking at each experimental site. The semantic differential (SD) method [32] used three pairs of adjectives on seven scales, including ‘comfortable to uncomfortable’ , ‘natural to artificial’ , and ‘relaxed to awakening’. The Profile of Mood State (POMS) scores were determined for the following six subscales: ‘tension-anxiety’ , ‘depression’ , ‘anger-hostility’ , ‘fatigue’ , ‘confusion’ , and ‘vigor’. A short form of the POMS with 30 questions was used to decrease the burden on the subjects [33-35]. The State-Trait Anxiety Inventory (STAI) [36] was used to evaluate anxiety.

A paired *t*-test was used to compare the mean physiological parameters between the two walking sites. The Wilcoxon signed-rank test was used to analyze differences in psychological indices reported after walking in the two environments. All statistical analyses were performed using SPSS 20.0 (SPSS Inc, Chicago, IL, USA). For all cases, *P* < 0.05 (one sided) was considered statistically significant.

## Results

**Figure 2:**
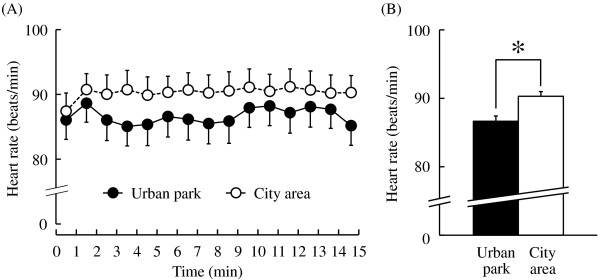
**The one-minute averages and the overall mean heart rate during the urban park walk and the city area walk. (A)** Changes in each 1-minute average heart rate over the 15-minute walk. **(B)** Overall mean heart rates. N = 12, mean ± standard error. *P < 0.05 determined by the paired *t*-test.

The subjects exhibited significant differences in physiological responses during a fifteen-minute walk in two distinct environments, an urban park and a nearby city area. The mean baseline heart rate did not differ significantly between sites before the walk (urban park: 87.5 ± 3.1 bpm (mean ± standard error), city area: 86.1 ± 2.9 bpm; *P* > 0.05); however, all mean heart rate values within one-minute epochs were lower during the urban park walk compared with those during the city walk (Figure 2A). The mean heart rate over the entire 15-minute period was significantly lower (by 4.0%) during the urban park walk than during the city walk (urban park: 86.7 ± 2.9 bpm, city area: 90.3 ± 2.6 bpm; *P* < 0.05; Figure 2B).

**Figure 3:**
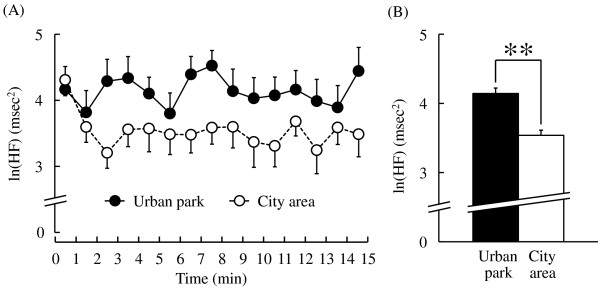
**The one-minute averages and the overall mean ln(HF) value of heart rate variability (HRV) during the urban park walk and the city area walk. (A)** Change in each one-minute ln(HF) value. **(B)** Overall mean ln(HF) values. N = 12, mean ± standard error. **P < 0.01, determined by the paired *t*-test.

**Figure 4:**
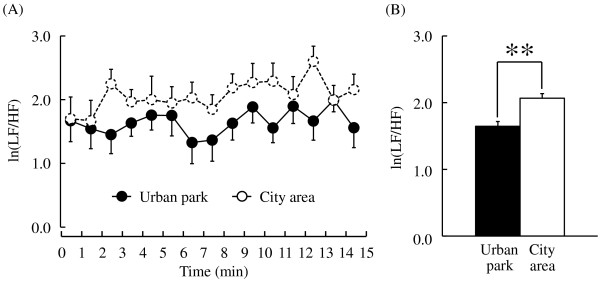
**The one-minute averages and the overall mean ln(LF/HF) value of heart rate variability (HRV) during the urban park walk and the city area walk. (A)** Change in each one-minute ln(LF/HF) value. **(B)** Overall mean ln(LF/HF) values. N = 12, mean ± standard error. **P < 0.01, determined by the paired *t*-test.

In addition, there were significant differences in HRV between the two sites. The mean normalized high-frequency (HF) component (ln(HF)), an estimate of parasympathetic nervous activity, was not significantly different at the start of the walk (urban park: 4.0 ± 0.2 msec^2^, city area: 4.3 ± 0.3 msec^2^; *P* > 0.05), while most mean ln(HF) values within one-minute epochs were higher during the urban park walk than during the city walk (Figure 3A). For the entire 15-minute duration, ln(HF) was 17.1% higher during the urban park walk than during the city area walk (urban park: 4.1 ± 0.2 msec^2^, city area: 3.5 ± 0.2 msec^2^; *P* < 0.01; Figure 3B). In contrast, the natural logarithm of LF/HF (ln(LF/HF)), an estimate of sympathetic nervous activity, was lower for most one-minute epochs during the urban park walk (Figure 4A), while the average ln(LF/HF) over 15 minutes was 20.3% lower than that for the city walk (urban park: 1.65 ± 0.18, city area: 2.07 ± 0.18, *P* < 0.01; Figure 4B). Again, there was no significant difference in baseline ln(LF/HF) values between sites (urban park: 2.14 ± 0.23, city area: 1.67 ± 0.35; *P* > 0.05).

**Figure 5:**
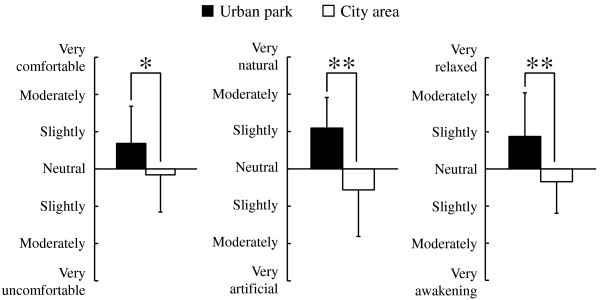
**Comparison of subjective scoring for ‘comfortable’, ‘natural’, and ‘relaxed’ feelings between the two environments according to the semantic differential (SD) method.** N = 17, mean ± standard deviation. *P < 0.05, **P < 0.01, determined by the Wilcoxon signed-rank test.

**Figure 6:**
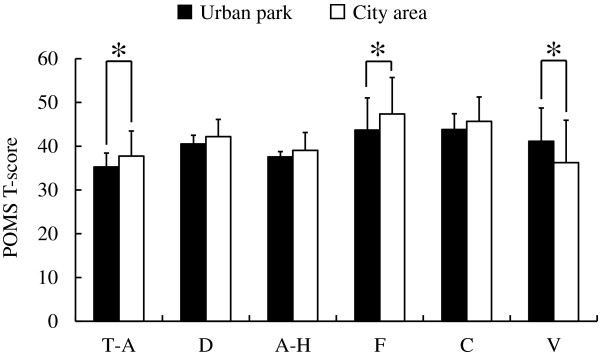
**Comparison of subjective Profile of Mood State (POMS) scores between the two environments.** T-A, tension-anxiety; D, depression; A-H, anger-hostility; F, fatigue; C, confusion; V, vigor. N = 17, mean ± standard deviation. *P < 0.05, determined by the Wilcoxon signed-rank test.

**Figure 7:**
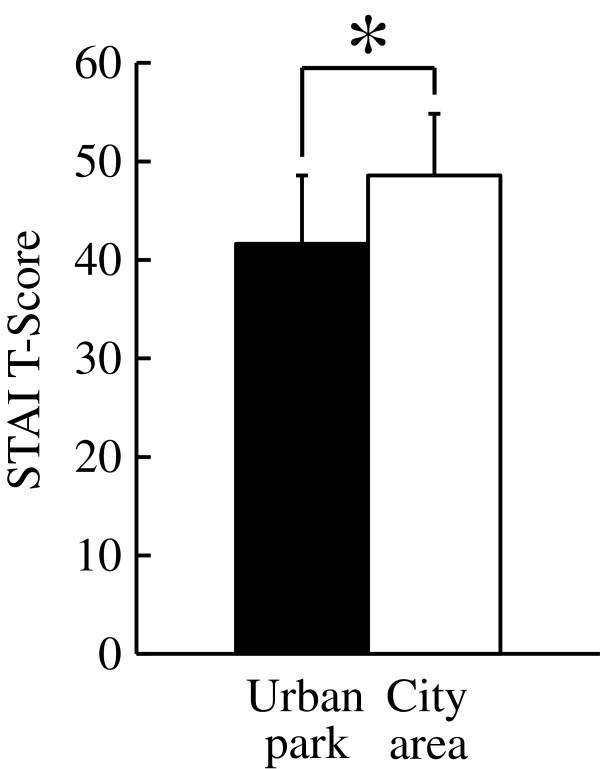
**Comparison of subjective State-Trait Anxiety Inventory (STAI) scores between the two environments.** N = 17, mean ± standard deviation. *P < 0.05, determined by the Wilcoxon signed-rank test.

Analysis of responses to three questionnaires completed after the urban park and city area walks, the SD method, the POMS scores, and the STAI scores revealed differences in psychological responses between the two environments. Significantly higher SD scores were observed following the urban park walk compared with those following the city walk for the following three adjectives: ‘comfortable’ , ‘natural’ , and ‘relaxed’ (*P* < 0.05; Figure 5). Differences were also detected in the POMS test (Figure 6), with scores for the negative subscales of ‘tension-anxiety’ and ‘fatigue’ being significantly lower after walking in the urban park than after walking in the city area (*P* < 0.05). Conversely, the positive mood state ‘vigor’ was significantly higher after the urban park walk (*P* < 0.05). There were no significant differences in the scores for ‘depression’ , ‘anger-hostility’ , and ‘confusion’. Finally, the total STAI score was 14.3% lower after the urban park walk compared with that after the city area walk (urban park: 41.6. ± 7.0 (mean ± standard deviation), city area: 48.6 ± 6.3; *P* < 0.05; Figure 7).

## Discussion

Access to urban green spaces may have significant physiological and psychological effects on urban residents.

Compared with those after a brief walk in the city area, the heart rate was significantly lower (-4.0%), parasympathetic nervous activity was enhanced (17.1%), and sympathetic nervous activity was suppressed (-20.3%) during a brief walk in the urban park. These results are consistent with those from previous studies on physiological responses to forest settings [9,11-15], suggesting that even small natural areas within a larger urban area can confer similar health benefits. These same HRV responses are often detected during massage [37,38] or yoga [39], so walking in an urban park may be a simple, accessible, and cost-effective method to improve general cardiovascular and mental health.

According to the analysis of the three questionnaires, the subjects in this study felt more ‘comfortable’ , ‘natural’ , ‘relaxed’ , and ‘vigorous’ after a walk in the urban park. In addition, negative emotions and anxiety were significantly lower after the urban park walk. These results on the psychological benefits of walking in an urban park are partly consistent with previous findings [5,9-12,15]. Because mental health is considered to be important in modern times [40], the psychological benefits of urban green space are expected to play a very important role in the promotion of mental health.

In a previous study, we examined the effects of urban green space in winter using the same experimental design and locations [28]. The current results corroborate our previous findings with one notable difference. In winter, a significant difference in sympathetic nervous activity was not detected between sites, while all other parameters (parasympathetic nervous activity, heart rate, and psychological indices) showed similar differences between walking environments.

Although we are now living in largely artificial urban environments, our physiological functions evolved in the natural environment [3,20,41]. The human tendency to seek natural environments implies that contact with nature may be important for promoting human health and well being [42]. The beneficial effects of urban green space suggest a simple accessible pathway to improved health. Furthermore, urban planners should pay more attention to maintaining and increasing accessible greenery in urban areas to improve the quality of life of the residents.

These findings provide empirical evidence for the physiological and psychological benefits of brief walks in an urban park. However, these results cannot be extrapolated to the female population or to other age groups because only 17 young male adults participated in this study. To generalize the findings, further studies based on larger and more heterogeneous cohorts are required.

Identification of differences in physiological states between natural and artificial environments is a critical issue in physiological anthropology, given the increasing disconnect between our evolutionary history and the modern living environment.

## Conclusions

The physiological and psychological responses elicited by this field experiment provide evidence for the physiological and psychological benefits of urban green space. A brief spring-time walk in an urban park shifted sympathetic/parasympathetic balance and improved mood state.

## Abbreviations

HF: high frequency; HRV: heart rate variability; LF: low frequency; POMS: Profile of Mood States; SD: semantic differential; STAI: State-Trait Anxiety Inventory.

## Competing interests

The authors declare that they have no competing interests.

## Authors’ contributions

CS contributed to the experimental design, data acquisition, statistical analysis, interpretation of results, and manuscript preparation. HI and MI contributed to data acquisition and statistical analysis. MM and MT participated in study design and data interpretation. YM contributed to the study design, interpretation of results, and manuscript preparation. All authors contributed to the preparation of this manuscript and are responsible for the final version.

## References

[B1] Kaplan R Kaplan S The Experience of Nature: A Psychological Perspective 1989 New York: Cambridge University Press

[B2] Kaplan S The restorative benefits of nature: toward an integrative framework J Environ Psychol 1995 15 169 182 10.1016/0272-4944(95)90001-2

[B3] Ulrich RS Simons RF Losito BD Fiorito E Miles MA Zelson M Stress recovery during exposure to natural and urban environments J Environ Psychol 1991 11 201 230 10.1016/S0272-4944(05)80184-7

[B4] van den Berg AE Koole SL van der Wulp NY Environment preference and restoration: (how) are they related? J Environ Psychol 2003 23 135 146 10.1016/S0272-4944(02)00111-1

[B5] Park BJ Furuya K Kasetani T Takayama N Kagawa T Miyazaki Y Relationship between psychological responses and physical environments in forest settings Landsc Urban Plan 2011 102 24 32 10.1016/j.landurbplan.2011.03.005

[B6] Groenewegen PG van den Berg AE de Vries S Verheij RA Vitamin G: effects of green space on health, well-being, and social safety BMC Public Health 2006 6 1 9 10.1186/1471-2458-6-1 16759375 PMC1513565

[B7] Shin WS Yeoun PS Yoo RW Shin CS Forest experience and psychological health benefits: the state of the art and future prospect in Korea Environ Health Prev Med 2010 15 38 47 10.1007/s12199-009-0114-9 19844774 PMC2793345

[B8] Shin WS Shin CS Yeoun PS Kim JJ The influence of interaction with forest on cognitive function Scan J Forest Res 2011 26 595 598 10.1080/02827581.2011.585996

[B9] Park BJ Tsunetsugu Y Kasetani T Morikawa T Kagawa T Miyazaki Y Physiological effects of forest recreation in a young conifer forest in Hinokage town, Japan Silva Fenn 2009 43 291 301

[B10] Lee J Park BJ Tsunetsugu Y Kagawa T Miyazaki Y Restorative effects of viewing real forest landscapes, based on a comparison with urban landscapes Scand J Forest Res 2009 24 227 234 10.1080/02827580902903341

[B11] Tsunetsugu Y Park BJ Ishii H Hirano H Kagawa T Miyazaki Y Physiological effects of Shinrin-yoku (taking in the atmosphere of the forest) in an old-growth broadleaf forest in Yamagata prefecture, Japan J Physiol Anthropol 2007 26 135 142 10.2114/jpa2.26.135 17435356

[B12] Tsunetsugu Y Lee J Park BJ Tyrvainen L Kagawa T Miyazaki Y Physiological and psychological effects of viewing urban forest landscapes assessed by multiple measurement Landsc Urban Plan 2013 113 90 93

[B13] Park BJ Tsunetsugu Y Lee J Kagawa T Miyazaki Y Li Q Effect of the forest environment on physiological relaxation using the results of field tests at 35 sites throughout Japan Forest Medicine 2012 New York: Nova 55 65

[B14] Park BJ Tsunetsugu Y Ishii H Furuhashi S Hirano H Kagawa T Miyazaki Y Physiological effects of Shinrin-yoku (taking in the atmosphere of the forest) in a mixed forest in Shinano Town, Japan Scan J Forest Res 2008 23 278 283 10.1080/02827580802055978

[B15] Lee J Park BJ Tsunetsugu Y Ohira T Kagawa T Miyazaki Y Effect of forest bathing on physiological and psychological responses in young Japanese male subjects Public Health 2011 125 93 100 10.1016/j.puhe.2010.09.005 21288543

[B16] Park BJ Tsunetsugu Y Kasetani T Hirano H Kagawa T Sato M Miyazaki Y Physiological effects of Shinrin-yoku (taking in the atmosphere of the forest) - using salivary cortisol and cerebral activity as indicators J Physiol Anthropol 2007 26 123 128 10.2114/jpa2.26.123 17435354

[B17] Li Q Morimoto K Nakadai A Inagaki H Katsumata M Shimizu T Hirata Y Hirata K Suzuki H Miyazaki Y Kagawa T Koyama Y Ohira T Takayama N Krensky AM Kawada T Forest bathing enhances human natural killer activity and expression of anti-cancer proteins Int J Immunopathol Pharmacol 2007 20 3 8 17903349 10.1177/03946320070200S202

[B18] Li Q Morimoto K Kobayashi M Inagaki H Katsumata M Hirata Y Hirata K Suzuki H Li YJ Wakayama Y Kawada T Park BJ Ohira T Matsui N Kagawa T Miyazaki Y Krensky AM Visiting a forest, but not a city, increases human natural killer activity and expression of anti-cancer proteins Int J Immunopathol Pharmacol 2008 21 117 127 18336737 10.1177/039463200802100113

[B19] Li Q Morimoto K Kobayashi M Inagaki H Katsumata M Hirata Y Hirata K Shimizu T Li YJ Wakayama Y Kawada T Ohira T Takayama N Kagawa T Miyazaki Y A forest bathing trip increases human natural killer activity and expression of anti-cancer proteins in female subjects J Biol Regul Homeost Agents 2008 22 45 55 18394317

[B20] Lee J Li Q Tyrväinen L Tsunetsugu Y Park BJ Kagawa T Miyazaki Y Maddock JR Nature therapy and preventive medicine Public Health-Social and Behavioral Health 2012 Rijeka: InTech 325 350

[B21] Dwyer JF McPherson EG Schroeder HW Rowntree RA Assessing the benefits and costs of the urban forest J Arboric 1992 18 227 234

[B22] Chiesura A The role of urban parks for the sustainable city Landsc Urban Plan 2004 68 129 138 10.1016/j.landurbplan.2003.08.003

[B23] Maas J Verheij RA Groenewegen PP Vries SD Spreeuwenberg P Green space, urbanity, and health: how strong is the relation? J Epidemiol Community Health 2006 60 587 592 10.1136/jech.2005.043125 16790830 PMC2566234

[B24] Mitchell R Popham F Effect of exposure to natural environment on health inequalities: an observational population study Lancet 2008 372 1655 1660 10.1016/S0140-6736(08)61689-X 18994663

[B25] Takano T Nakamura K Watanabe M Urban residential environments and senior citizens’ longevity in megacity areas: the importance of walkable green spaces J Epidemiol Community Health 2002 56 913 918 10.1136/jech.56.12.913 12461111 PMC1756988

[B26] Dye C Health and urban living Science 2008 319 766 769 10.1126/science.1150198 18258905

[B27] Lee ACK Maheswaran R The health benefits of urban green spaces: a review of the evidence J Public Health 2010 33 212 222 10.1093/pubmed/fdq068 20833671

[B28] Song C Joung D Ikei H Igarashi M Aga M Park BJ Miwa M Takagaki M Miyazaki Y Physiological and psychological effects of walking on young males in urban parks in winter J Physiol Anthropol 2013 32 18 10.1186/1880-6805-32-18 24168929 PMC3817995

[B29] Task Force of the European Society of Cardiology and the North American Society of Pacing and Electrophysiology Heart rate variability: standards of measurement, physiological interpretation and clinical use Circulation 1996 93 1043 1065 10.1161/01.CIR.93.5.1043 8598068

[B30] Pagani M Lombardi F Guzzetti S Rimoldi O Furlan R Pizzinelli P Sandrone G Malfatto G Dell’Orto S Piccaluga E Power spectral analysis of heart rate and arterial pressure variabilities as a marker of sympatho-vagal interaction in man and conscious dog Circ Res 1986 59 178 193 10.1161/01.RES.59.2.178 2874900

[B31] Kobayashi H Park BJ Miyazaki Y Normative references of heart rate variability and salivary alpha-amylase in a healthy young male population J Physiol Anthropol 2012 31 9 10.1186/1880-6805-31-9 22738348 PMC3384356

[B32] Osgood CE Suci GJ Tannenbaum P The Measurement of Meaning 1957 Urbana, IL: University of Illinois Press

[B33] McNair DM Lorr M An analysis of mood in neurotics J Abnorm Soc Psychol 1964 69 620 627 10.1037/h0040902 14241709

[B34] McNair D Lorr M Droppleman L Profile of Mood States Manual 1992 San Diego, CA: Educational and Industrial Testing Services

[B35] Yokoyama K POMS Shortened Version-Manual and Commentary on Cases 2005 Tokyo: Kaneko Syoboh [in Japanese]

[B36] Hidano N Fukuhara M Iwawaki M Soga S Spielberger CD State-Trait Anxiety Inventory-Form JYZ 2000 Tokyo: Jitsumu-Kyoiku Syuppan [in Japanese]

[B37] Delaney JP Leong KS Watkins A Brodie D The short-term effects of myofascial trigger point massage therapy on cardiac autonomic tone in healthy subjects J Adv Nurs 2002 37 364 371 10.1046/j.1365-2648.2002.02103.x 11872106

[B38] Buttagat V Eungpinichpong W Chatchawan U Kharmwan S The immediate effects of traditional Thai massage on heart rate variability and stress-related parameters in patients with back pain associated with myofascial trigger points J Bodyw Mov Ther 2011 15 15 23 10.1016/j.jbmt.2009.06.005 21147414

[B39] Shapiro D Cook IA Davydov DM Ottaviani C Leuchter AF Abrams M Yoga as complementary of depression: effects of traits and moods on treatment outcome Evid Based Complement Alternat Med 2007 4 493 502 10.1093/ecam/nel114 18227917 PMC2176141

[B40] Murray CJL Lopez AD Evidence-based health policy - lessons from the global burden of disease study Science 1996 274 740 743 10.1126/science.274.5288.740 8966556

[B41] Miyazaki Y Park BJ Lee J Osaki M, Braimoh A, Nakagami K Nature therapy Designing our Future: Local Perspectives on Bioproduction, Ecosystems and Humanity 2011 New York, NY: United Nations University Press 407 412

[B42] Frumkin H Beyond toxicity, human health, and the natural environment Am J Prev Med 2001 20 234 240 10.1016/S0749-3797(00)00317-2 11275453

